# A conceptual framework of game-informed principles for health professions education

**DOI:** 10.1186/s41077-016-0030-1

**Published:** 2016-11-17

**Authors:** Rachel H. Ellaway

**Affiliations:** University of Calgary, Alberta, Canada

**Keywords:** Video Game, Instructional Design, Game Design, Board Game, Health Profession Education

## Abstract

Games have been used for training purposes for many years, but their use remains somewhat underdeveloped and under-theorized in health professional education. This paper considers the basis for using serious games (games that have an explicit educational purpose) in health professional education in terms of their underlying concepts and design principles. These principles can be understood as a series of game facets: competition and conflict, chance and luck, experience and performance, simulation and make-believe, tactics and strategies, media, symbols and actions, and complexity and difficulty. Games are distinct and bound in ways that other health professional education activities are not. The differences between games and simulation can be understood in terms of the interconnected concepts of isomorphism (convergence with real-world practice) and anisomorphism (divergence from real-world practice). Gaming facets can extend the instructional design repertoire in health professional education.

## Background

A “serious game” is a game that has “an explicit and carefully thought-out educational purpose” [1]. There has been a growing interest in recent years in the educational use of serious games in both higher education [2] and in health professions education [3–5]. While there is a long history of games being used for training purposes in other professions (such as management and the military [1]), games have not yet become a part of the core repertoire for training health professionals. Attempts to resolve uncertainty about the place of serious games in health professions education through evidence synthesis have described the problem but have not necessarily helped to clarify matters. For instance, although they provide many examples of the use of games in the training of health professionals, both Smith [6] and Akl et al. [7] concluded that games are not intrinsically good or bad, their efficacy is highly context-bound, and even when they are shown to be effective, there are still questions as to whether they are efficient given their associated development costs. Similarly, Graafland et al. observed that, although there are studies that show that certain kinds of games can be used to positively effect in health professional education, the evidence base for this is somewhat lacking [5].

Rather than simply repeating these conclusions or giving yet more examples of games in health professions education (HPE), I will instead consider the basis for using serious gaming as a source of concepts and techniques to guide educational practice and to encourage critical engagement with and a more in-depth consideration of games and gaming in the field of health professions education. To that end, I will set out a faceted framework of game attributes, differentiate between gaming and simulation, and describe some of these game facets’ educational affordances. I have provided a glossary of terms used in this paper as an aid to grounding this work.

## Games and gaming

I will start with the general phenomena of “play” and “games”. The young of many mammalian species engage in activities that are considered to be play. By doing things individually and collectively that approximate to adult “serious” activities, they develop adult skills without having to deal with the consequences of these activities while they are still learning. To that end, play is not simply a diversion or a reflection of immaturity: “play is essential to development… [it] allows children to use their creativity while developing their imagination, dexterity and physical, cognitive and emotional strength” [8]. Given that play is intrinsically linked to early learning it is not unreasonable for us to seek to leverage these potential advantages for professional educational purposes.

Play and games share a number of features; they are both abstracted from the real world, and they depend on a psychosocial moratorium where reality is willingly suspended, thereby allowing participants to explore alternative and new ways of being and acting. To suspend reality and accept an alternative way of thinking and being is to play or game, and for reality to intrude is to fall back to a quotidian reality. Both play and games can be individual or social activities, and they both involve abstraction in terms of selecting from reality or imagination what elements they will (and will not) include.

However, and despite these similarities, there are essential differences between them: “play is an open-ended territory in which make-believe and world-building are crucial factors; games are confined areas that challenge the interpretation and optimizing of rules and tactics” [9]. While play is not expected to lead to any particular outcome (other than being pleasurable or distracting), a game is bound by goals, rules, and challenges. To play a game is to accept its rules and challenges, while to engage in play is to change boundaries at will or to do without them altogether [10]. Boundaries are therefore a critical component of games. This is reflected in the heinous crime of cheating; an intentional and tacit transgressing of a game’s boundaries for personal advantage. Play on the other hand has little or no concept of cheating.

We should also differentiate between game templates (from which any number of game instances can be run) and specific game instances (time- and context-bound instantiations of a game template). For example, the game template of chess specifies the board, the pieces, the allowed moves for each piece, the limits of play (two opponents alternating moves between them), the rules of check, the objective of seeking to place the opponent in checkmate, and the three possible end points of conceding, stalemate, or checkmate. From this simple template, a vast number of game instances can be played, as the rich tradition of chess game writing illustrates. However, although the game template establishes the boundaries for a game instance, it does not determine how an instance will unravel. Without some form of emergence, a game is simply an algorithm that can be learned. Indeed, perceiving an underlying algorithm in education and attempting to play it rather than the task at hand has parallels in the rather negative concept of “surface learning” [11].

### Caillois’ principles

Although games share some common high-level properties, there are many different kinds of games. Caillois defined four aspects of games (paraphrased and expanded on here) [12]:

- *Agon* (competition or conflict) is fundamental to a game’s objectives, and it can be realized in many different ways. We can differentiate between competition (improving performance) within a game instance (similar to “reflection in action”) and competition between game instances (similar to “reflection on action”). In the context of games, competition is usually continuous and creative while conflict is episodic and tends to focus on mitigating harm or resolving the basis for conflict. For instance, a player or team may seek to improve on their own performance, to do better than others (either synchronously or asynchronously), or to master the game or its objectives. Improved performances may be realized by attempting to play the game faster, longer, with greater dexterity, with higher scores, with fewer mistakes, or with fewer penalties.
- *Alea* (chance or luck) may be realized in terms of a game instance’s starting conditions, by the actions of other players or by the deliberate introduction of random elements. The use of *alea* can keep a game fresh and challenging even after playing it several times. For instance, the board game “The Last Straw” (www.thelaststraw.ca) was designed to help players learn about social determinants of health by having each player rolling a dice to randomly select what factors will impact their character’s life expectancy; in this way every game instance is different.
- *Ilinx* is realized in the form of either physical experiences (such as haptic feedback) or physical performance (such as skill in manipulating a physical object like a ball or a scalpel). For instance, the Nobel Prize blood typing game (www.nobelprize.org/educational/medicine/bloodtypinggame/) requires a player to draw blood, test it, and then give blood by manipulating onscreen icons. However, given that clinical practice involves a complex interplay of cognitive and physical activity, we can expand on Caillois’ concept of *ilinx* to include cognitive as well as physical dimensions both of experience (contextual, circumstantial) and performance (active and required by the game).
- *Mimicry* is about the use of simulation or make-believe in a game. This can be realized through the use of scenarios, role-playing, or other reflections of real or imagined worlds. I will return to this aspect of games later in this paper.

We can reasonably expect that many, if not most, serious games will embody more than one of these principles rather than conforming to one type to the exclusion of the others. We should therefore treat this as a faceted typology (rather than a taxonomic one) [13]. The generative nature of a faceted model can represent both the plurality of existing game designs as well as new types based on the same base facet repertoire. We can therefore consider the patterns of games, their similar but not necessarily identical attributes. Such an approach allows for greater flexibility in designing and appraising games. A faceted model also reflects the aspiration of “game-informed learning” where educators employ aspects of games in educational activities that do not have to be explicitly game-like [14]. A series of core game facets is set out in Table 1.

**Table 1:** A comparison of component facets for games and simulation

Facet	Realized in games…	Realized in simulation…
Competition	Almost always present in some form or other, structured through win/lose states, rankings, scores, progression through levels	Depends on the scenario but generally limited use of competition other than seeking to improve performance over time or in the context of simulation for assessment purposes
Conflict	Either isomorphic such as in war games, anisomorphic (in terms of a player’s symbolic relationships with their game opponents), or absent altogether in opponent-free games such as puzzles	Only present if it is isomorphic with practice: conflict in communication, management, teamwork, etc. Conflict may be realized in the medical problem or challenge or in the relationships between participants
Chance/luck	Wide range from isomorphic to anisomorphic —typically in the form of random elements (dice or cards) to player responses (chess, go)	Depends on scenario—may be realized in randomized patient data, randomized pathways through algorithms, or interactions with other participants
Experience	Wide range from isomorphic to anisomorphic—linked to game media	Always present in some form or other in physical simulation, limited in onscreen simulation
Performance	Wide range from isomorphic to anisomorphic—usually linked to the game medium employed	Typically in the form of clinical skills in physical simulation, extremely limited in onscreen simulation
Simulation	Wide range from isomorphic to anisomorphic	Direct representations of clinical settings, patients, presentations, tasks, challenges
Make-believe	Wide range of uses from isomorphic (conformance with mythic or fantasy idiom) to anisomorphic (innovative)	Fictional or fictionalized narratives and roles. Fourth-wall techniques such as debriefing in role and providing feedback out of role
Tactics and strategies	Wide range from isomorphic to anisomorphic	Depends on scenario—should be isomorphic with real-world practice
Media	Physical, virtual, or augmented	Physical, virtual, or augmented
Symbols and actions	Tendency to greater abstraction (anisomorphism)	Depends on scenario—should be isomorphic with real-world practice
Complexity	Game-specific, represented in the game’s boundaries	Depends on scenario and its intended outcomes—should be isomorphic with real-world practice
Difficulty	Depends on game levels and/or opponents	Should relate to intended outcomes and transfer to professional practice

### Building on Caillois’ typology

If we consider the essential game facets, we can expand on Caillois’ model to consider other dimensions or features (also set out in Table 1):

- Tactics and strategies a game requires or allows. Games of chance (*alea*) are about dealing with what is (sometimes literally) dealt to you, whereas this facet is about players’ expected or allowed responses to the emerging challenges of *alea*, the options and the requirement for players to try different tactics and/or different strategies within a game instance. For instance, the card games of bridge and whist have similar rules and gameplay but require very different kinds of tactical and strategic thinking. In HPE, some virtual patients present game-like activities that are about reasoning within a fixed “medical model” while others may be more free-form in nature [15].
- The media through which a game is realized. There are some highly recognizable and traditional (perhaps even archetypal) game media (such as cards, dice, and scores) and formats (such as board games, TV-style quizzes, and first-person shooter video games). Games may involve manipulating physical objects or virtual (onscreen) objects or a combination of both (such as augmented reality games like Pokemon Go). While many games can be rendered in either physical or virtual formats (such as chess, card games, or board games like Scrabble), the game medium can make or break player experiences and therefore the educational affordances of those experiences. For instance, there are significant limitations to how video games can represent the intricacies of clinical practice, which limits their utility in affording educational experiences [16].
- Symbols and actions. Games may simulate real-world practice, but, in doing so, they inevitably emphasize some aspects over others, they omit other aspects, and they may add new ones. Peters et al. [17] reflect this in their description of game design as a process of reduction (keeping only that which is relevant to game play or the game’s objectives), abstraction (reducing complexity to enable a rule system), and symbolization (creating a “new symbolic structure”). The latter includes permissible paths within the game, the order and type of play, and the availability and agency of game artifacts. We can also consider issues of scale: the activity as a whole (objectives), the specific ways in which the broad objectives are to be addressed (such as moves or turns in a game), and the stepwise processes that go to make up the actions (such as rolling a dice or controlling a game avatar) [18, 19]. The balance between simulation and abstraction is a critical one in game design, and I will revisit it in the next section.
- Complexity and difficulty. Games differ in how adaptable they are to different players’ abilities and strategies and how and to what extent a game helps players to learn from and within the game, for instance, by providing players with feedback, hints, and other kinds of guidance and support. Caillois described the structural complexity of games by differentiating between “*paidea*” (minimally organized games) and “*ludus*” (highly organized games) [12]. We might, for instance, say that a role-playing game reflects *paidea* while a game (such as chess) that focuses on iterative acquisition of mastery of a particular technique is a form of *ludus*.

These facets can individually determine the design and the evaluation of games, with the potential to guide instruction design. For instance, the use of role-play or collaboration may be gainfully employed in a simulation scenario without worrying whether or not the resulting activity is (or is not) a game; its educational efficacy is what counts.

## Serious games and instructional design

Serious games differ from other kinds of games by having “an explicit and carefully thought-out educational purpose” [1]. It is important therefore to consider the educational affordances of serious games (how they facilitate learning) as well as their outcomes (what they help the learner or teacher to achieve). For instance, games may be used to increase learner engagement [20, 21] or to facilitate transfer from learning to practice [6, 22]. Indeed, while there are good motivational reasons for employing games or elements of games in HPE (typically as a means of increasing learner engagement), transfer to practice is perhaps the more important goal (at least in the context of this journal), and as such, we return to the principle of simulation in games for HPE.

Given the similarities between simulation and games, it is not surprising that there is some debate about whether games and simulations are discrete phenomena [23, 24]. In the healthcare education context: “when … gaming characteristics [such] as conflict, players, rules and payoff are combined with simulation, they result in the representation of a particular decision making process. Only those games that depict reality in precise and regulated ways are simulations” [23]. However, it may be that the reliable depiction of reality is not the essential difference between games and simulation (at least in the context of simulation-based learning), not least because serious games and simulation-based learning seemingly overlap in many ways. We need therefore to consider the translation between games and professional practice a little more carefully.

### Validity and isomorphism

We can explore the connections between game-playing and professional practice in terms of the interlinked concepts of validity and isomorphism. Raser described game validity in terms of four dimensions: psychological reality (“behaviors in the game correspond to behaviors in the reference system”), structural validity (theory and assumptions are isomorphic between game and reference system), process validity (processes are isomorphic between game and reference system), and predictive validity (outcomes are isomorphic between game and reference system) [25]. Isomorphism in the context of serious games refers to the convergence between structure, form, and performance in a game and its real-world referents. The isomorphic referent does not necessarily have to be the simulated world, it may also be normative (drawing on a standard repertoire, for instance using the Jeopardy format for a quiz), coercive (responding to constraints such as curricular objectives or the will of professional communities), or mimetic (imitation to confer legitimacy in the face of uncertainty) [26]. These differences seem less to be at the category level of simulation and gaming and more at the component level. We can therefore make expand on this to compare games and simulation in terms of the game facets described earlier (see Table 1).

Given that the goal of health professional education is about preparing for future professional practice, a serious game used in its service should contribute to this goal. Isomorphism is a key concept in establishing the link between serious games and professional practice, both in terms of convergence and divergence. For instance, we can contrast *simulation* (isomorphism) with *make-believe* and abstraction (as two forms of anisomorphism). Both have a role to play in serious games; simulation enables transfer to practice, and make-believe permits the use of narrative and theatrical elements (such as contrived backstories and scenarios, role-play, and fourth-wall techniques such as time-outs) to create and sustain the game experience.

It is important to note, however, that the relevance of isomorphism in serious games is not just about convergence; it is also about deliberate divergence from reality; indeed, games require the reduction, abstraction, and symbolization of reality [17]. Deciding which aspects of reality are to be altered is one of the most critical steps in constructing a game, serious or otherwise. For instance, anisomorphism in a game to develop junior health professional students’ clinical reasoning skills would involve simplifying and focusing the challenges and options they face to reflect their capabilities and to encourage learning. The use of anisomorphism in this case can be linked to the development of expertise [27] and to the deliberate scaffolding of learning experiences [28]. To understand a serious game then is to appreciate both how it converges with real-world practice and how it diverges from it.

### Educational affordances

Both isomorphism and anisomorphism have educational uses and, in the case of serious games, should be selected in a configuration that can best help learners to converge on the intended educational objectives. Indeed, each of the different game facets can afford different instructional or educational benefits, as outlined in Table 2. This reflects the central tenet of game-informed learning, that it is not games that are (or are not) educationally effective, it is the different facets of games that can confer educational advantage. However, as with any instructional modality, these advantages are not causal in effecting learning and the extent to which they are realized depends on a number of factors, including the perceptions of the participants and the relationships between playing a game and the broader educational program or environment within which this occurs.

**Table 2:** Serious game facets and their educational affordances

Facet	Educational affordances
Competition	Motivates learner to improve their performance by challenging them to make iterative changes to their approach to a task or situation guided by feedback and other performance indicators [12, 41, 47]
Conflict	Structured opportunities to learn how to deal with conflict—how to identify it, how to work when there is conflict (particularly when it disrupts collaboration between participants), and/or how to resolve conflict. Given the potential for stress, conflict involves higher cognitive load and is introduced only once a learner can handle a conflict-free situation [12, 42]
Chance/luck	Either simulation of chance factors in real-world practice (such as whether a patient has a particular condition or will respond to a particular treatment) or as a way of adding unpredictability to game play and thereby adding to the complexity and/or difficulty of a game [12, 15]
Experience	Increases immersion in the game (by making it more realistic or familiar), builds familiarity with particular kinds of situations, or augments the complexity and/or difficulty of a game [12, 47, 48]
Performance	To facilitate skill development (physical and cognitive) with direct transfer to practice and to rehearse and refine skills in different and emerging situations [12, 38, 39, 46]
Simulation	Depends on task related to simulators (things that simulate—media) and simulations (activities that simulate) [12, 34, 39, 49]
Make-believe	Allows participants to explore roles and situations, to develop their professional identities, and to develop empathy by “walking in someone else’s shoes” (psychosocial moratorium). The use of make-believe should be credible and align with practice situations, which in healthcare professional education is typically articulated in the form of case narratives and role profiles [12, 14, 38, 50]
Tactics and strategies	Should converge with those required in professional practice. Should not encourage “gaming” in the sense of misrepresenting performance by manipulating the underlying game boundaries at the sake of the intended learning outcomes [15, 51]
Media	Media choice depends on the task and the intended learning outcomes; it should align or converge with real-world professional practice [15, 16]
Symbols and actions	Should converge with professional practice, increasing isomorphism and reduced abstraction over time [17–19, 43]
Complexity	Should reflect scaffolding principles of increasing autonomy and convergence with real-world professional practice [38, 44, 45]
Difficulty	Should reflect scaffolding principles of increasing autonomy and convergence with real-world professional practice [38, 44, 45]

Not only is the use of different game facets in designing educational artifacts and activities a matter of selecting and combining them according to their instructional affordances, the design and use of serious games as a whole can be understood in terms of instructional design: “the process of deciding what methods of instruction are best for bringing about changes in student knowledge and skills for a specific course content and a specific student population” [29]. To that end we can map the elements of instructional design [30] to the instructional design affordances of serious games and game facets (see Table 3).

**Table 3:** Mapping the elements of instructional design [30] to the instructional design affordances of serious games and game facets

Elements of instructional design	Serious game affordances
Learners and learning processes	Games should be designed to be used. The design and use of serious games needs to align with learners’ needs, their capabilities, and their expectations. The focus should be on engineering compelling educational experiences.
Learning and performance contexts	Serious games should be:
	1) Appraised as learning and performance contexts in and of themselves
	2) Appraised in terms of their intended roles and impacts in the broader educational contexts in which they are used
	3) Appraised in terms of their facilitation of learning transfer to practice contexts
Content structure and sequence	Serious games can extend the instructional design repertoire by adding facets and templates for the creative and deliberate use of simulation and make-believe, symbols and symbolic actions, game boundaries and rules, and attention to learning experiences.
Instructional strategies	Serious games can extend the instructional design repertoire by adding facets and templates for the creative and deliberate use of competition and conflict, chance and luck, physical and cognitive performance, tactics and strategies, and complexity and difficulty.
Media and delivery systems	Gaming media can extend the instructional design repertoire, both by association (such as using the label of “game” for motivational reasons) and for their direct affordances (such as the use of multiplayer virtual worlds).
Designers and design processes	Serious games can present constructive challenges to instructional design norms and practices as well as extending the instructional designers’ repertoire.

**Fig. 1:**
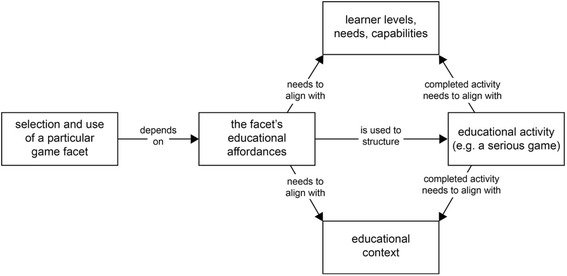
Cascade of instructional design decisions when making use of game facets

By mapping back and forward between serious games and instructional design principles and affordances, we can move to an understanding of serious games as a loosely bound category of educational activity designs that can have different educational uses based on the game facets they employ and on their alignment with learner needs and the educational contexts within which they work (see Fig. 1).

## Discussion

As much as serious games can afford instructional and motivational advantages, they are sometimes seen as rather immature or lacking in serious intent. This is reflected in a widespread, if tacit, sense that the use of games in health professions education does not reflect well on the gravitas of clinical practice and that to use them in this context requires us to clearly differentiate between serious and non-serious games. Unfortunately, this dichotomous perspective obscures the more important issue that “game” is a rather ill-defined aggregate of activity designs and components that can be realized in a multitude of ways, many of which already intersect with mainstream instructional strategies.

A central goal for this paper has been to encourage both a critical engagement with and a more in-depth consideration of games and gaming in the field of health professions education. Rather than listing game types or exploring them from a taxonomic perspective, I have set out a framework of game facets, I have used these facets to differentiate between gaming and simulation, and I have described them in terms of their educational affordances. In doing so, this kind of faceted approach can address the fundamental category problem of asking whether games are educationally effective. Different games employ different game facets in different ways; it is the educational avoidances of the facets that are used in any given instance that confer educational advantage. This reflects pattern-based faceted thinking [31] associated with other kinds of activity design such as PBL [32], team-based learning [33], and simulation [34]. Not only does this approach give more flexibility and precision in considering games for educational purposes, these facets can be understood as part of the broader instructional design repertoire for health professions education. After all, many of these facets are already to be found in health professions education activity designs. For instance, competition, whether with oneself or with others, is intrinsic to most contemporary assessment practices, and simulation is to be found almost everywhere within the field of health professions education [35].

It might be argued therefore that there is nothing particularly new about games and gaming in health professions education, and it is only the emphasis on certain facets or the particular configurations of facets that differentiates game activities from more mainstream practices in health professions education. However, the novelty of videogame, gamer culture, and the many devices that can be used to play games seems to have blinded us to the true nature of serious games and it has confused the debate over their use in health professional education. As educators, we need to make deliberate and informed use of game facets as part of our instructional design repertoire.

For instance, games are distinct and bound in ways that other health professional education activities are not. Games have clear success and failure criteria, they have specific and somewhat abstract rules, they depend on the use of structured feedback both within and between game instances, and they require participants to engage in the psychosocial moratoria of role-play, competition, and conflict. This suggests that games and game facets involve much larger (and potentially better) use of anisomorphism to create compelling experiences and drive learning than other activity types in the health professions education repertoire. It is an interesting paradox that learning in games can be driven by divergences from practice while mainstream thinking in simulation-based learning is so concerned with its convergence with professional practice.

In focusing on game facets and their instructional uses, I have perforce neglected other issues in and around the use of games in health professional education that are nevertheless deserving of attention. I will consider two of these briefly. Firstly, health professionals, in particular health professions learners, may be less experienced in gaming because of the effort required to build a credible profile to gain entry to a professional school. Because they tend to be relatively inexperienced in video games, they are likely to be less disposed to select them as a preferred modality of learning. It would be wrong therefore to make a generational assumption that younger people are intrinsically positively disposed towards the use of games. Secondly, perhaps the biggest elephant in the room is that of the economics of developing and using games in health professional education: “the time and skills required to set up such environments can still be a barrier to adoption as can the variability in student hardware and connectivity” [6]. However, the faceted approach would suggest that we should not assume that games will cost more in general. Answering economic questions depends on what facets are used, how they are combined, and how they are realized in a game template.

It is also important to note that this paper differs from other theoretical perspectives on serious games that have been proposed in fields outside of health professional education. Indeed, there have been many connections made between serious games and learning theories such as behaviorism, situated learning, transfer theory, and problem-based learning [36–38]. However, these have tended to focus on the alignment and fit between game and theory rather than on the faceted approach I have presented in this paper. Where game facets or elements have been considered, attention has tended to focus on the differences between game and pedagogical elements rather than on their educational affordances [39].

This reflects a more extensive consideration of educational games in education as a whole than there has in health professions education, with work in the latter tending to focus on particular games and their applications with the assumption that they have intrinsic and beneficial educational affordances [7, 40]. There are some exceptions, for instance, the connections between games and activity theory have been considered within health professions education [18] as well as in other fields [19]. van Staalduinen’s exposition of game properties is particularly notable as it takes a similar faceted mapping approach to that set out in this paper [41]. While there are similarities between the two models in terms of common facets (such as conflict, rules, and feedback), van Staalduinen’s model concentrated on the more operational aspects of actual games (such as pieces, players, and communication) rather than on the broader principles set out in the typology advanced in this paper. A key difference then is whether we should focus on the specifics of things that are explicitly considered to be games or instead on game-informed principles that can be realized in activities that are not games per se. In the context of exploring the connections between game and simulation in support of health professions education, my argument has been that a game-informed approach is perhaps the more useful of the two.

I acknowledge some limitations to the work presented here. I have taken somewhat hypothetico-deductive approach in synthesizing and shaping key concepts around serious gaming and deriving a framework and set of game-informed principles for health professions education. In doing so, I have not presented an evaluation of this model, nor have I tested it robustly in practice. Indeed, it would be hard to do so in any comprehensive way given its broad reaching scope. I have also not undertaken an exhaustive audit of the literature on serious games from outside of health professions education. The many examples that I have drawn upon indicate a paucity of application to our field and its particular needs and dynamics that may account for the lack of uptake compared with other professional training paradigms. Indeed, taking an instructional design perspective (as presented here) has enabled a critical stance on both the advantages and limitations of different serious game facets that does talk to some of the key concerns within the field.

I started this essay with the observation that, while there have been many calls for, and champions of, games in health professions education, the category of “serious games” was broad and their educational affordances ill-defined. It would be easy then to suggest that the answer to this is more research into particular uses of serious games in training health professionals. However, as I have set out, the catechism of “more research is needed” is too simplistic, not least because there has already been much research in to the educational use of games; the long-running Medicine Meets Virtual Reality conference (www.nextmed.com) and the US military-funded TATRC program (www.tatrc.org) are just two indicators of this effort. Rather, the main lesson here is the need and opportunity to see past the current *sturm und drang* of video games and gaming to the advantages that game-informed learning can afford health professional education. A key implication of the model I have presented is to connect individual and collections of game facets to educational theory and evidence. I have not done so here as this is in itself a significant task as, while there are links with extant work, it is not in the context of game facets. For instance, topics such as transfer [38], competition [11], conflict [42], shared symbols [43], complexity [44], scaffolding [45], and performance [46] have been previously explored. A systematic exposition of these connections is nevertheless a logical next step in pursuing this line of inquiry, as is the exploration of those elements that have a less robust evidential basis such as the use of chance.

A judicious use of gaming facets has the potential to extend and enhance the instructional design repertoire in health professions education, and in doing so it can act as a challenge to orthodox thinking that would see the unit of instructional value as the game rather than as its different facets. Moreover, a clearer focus on different game facets allows for the exploration of broader issues such as the educational value of activities that diverge from real world practice as well as those that converge with it.

## Conclusion

Different game facets have different educational affordances, many of which are to be found in other kinds of activities. Rather than questioning whether or not things that are recognizably “games” should be used (or used more) in health professions education, we should be considering what it is about games that makes them useful or compelling educationally; to what extent and in what way different game facets are present or absent in our practice and research; and how they can be used to effect desirable educational outcomes. And yes, more work is needed in this area.

## Glossary

Anisomorphism: dissimilarity or divergence between activity in a game and its referent real-world activity. Anisomorphism can involve the fictionalizing or abstraction of real-world elements or the introduction of elements without real world referents.
Game: structured and bound activities that both converge on and diverge from real-world practice and that involve competition in different forms. Games may be used for entertainment and educational purposes.
Game facet: a design principle that can be realized in a game template or instance.
Game instance: time- and context-bound instantiations of a game template.
Game template: a specification from which any number of game instances can be run, includes rules and other boundaries.
Isomorphism: similarity or convergence between activity in a game and its referent real world activity.
Play: unstructured and improvised activities that involve competition in various forms as well as other aspects in various combinations. Play can be educational but not in a purposive way.
Serious game: a game that has “an explicit and carefully thought-out educational purpose” [1].
